# Adapting enzymes to improve their functionality in plants: why and how

**DOI:** 10.1042/BST20230532

**Published:** 2023-10-03

**Authors:** Edmar R. Oliveira-Filho, Cătălin Voiniciuc, Andrew D. Hanson

**Affiliations:** Horticultural Sciences Department, University of Florida, Gainesville, FL, U.S.A.

**Keywords:** agriculture, directed evolution, enzymology, plant breeding

## Abstract

Synthetic biology creates new metabolic processes and improves existing ones using engineered or natural enzymes. These enzymes are often sourced from cells that differ from those in the target plant organ with respect to, e.g. redox potential, effector levels, or proteostasis machinery. Non-native enzymes may thus need to be adapted to work well in their new plant context (‘plantized’) even if their specificity and kinetics *in vitro* are adequate. Hence there are two distinct ways in which an enzyme destined for use in plants can require improvement: In catalytic properties such as substrate and product specificity, *k*_cat_, and *K*_M_; and in general compatibility with the milieu of cells that express the enzyme. Continuous directed evolution systems can deliver both types of improvement and are so far the most broadly effective way to deliver the second type. Accordingly, in this review we provide a short account of continuous evolution methods, emphasizing the yeast OrthoRep system because of its suitability for plant applications. We then cover the down-to-earth and increasingly urgent issues of which enzymes and enzyme properties can — or cannot — be improved in theory, and which in practice are the best to target for crop improvement, i.e. those that are realistically improvable and important enough to warrant deploying continuous directed evolution. We take horticultural crops as examples because of the opportunities they present and to sharpen the focus.

## Why enzymes can need adapting for plant applications

Enzymes can need improving in two conceptually different ways: In *what* they do, and in *how well* they do it when expressed in plant cells. First, the ‘*what*’ facet of enzyme improvement: Building a synthetic metabolic pathway can require enzymes (‘parts’) with activities that do not exist in nature, at least as anything more than minor side-activities of other enzymes [1–3]. Furthermore, even when mediating their native reaction, most enzymes ‘are sloppy and mediocre’ [4] because they can use alternative substrates, form side-products, and have modest yet improvable *k*_cat_ and *K*_M_ values [5]. Hence, whichever organism an enzyme part is to operate in, its core properties may need optimizing. Besides substrate/product specificity and kinetics, these properties include feedback inhibition by endogenous metabolites [6], herbicide sensitivity [7], and vulnerability to self-inactivation as a consequence of catalysis [8].

Turning to the ‘*how well*’ facet: If an enzyme is to operate optimally, or even at all, in a plant cell it must be compatible with that cell's physicochemical and biochemical environment, e.g. the temperature, oxygen tension, redox potential, osmolarity, small-molecule effector concentrations, and protein degradation system [9]. These parameters and others vary between organisms and most can vary between tissues in the same plant. Many enzymes used in plant synthetic metabolism designs, either ‘as-is’ or after engineering, are sourced from outside the plant kingdom or from taxa, tissues, or cellular compartments quite different from the chassis they are put into or destined for. Typical examples are: Photorespiratory bypasses in tobacco chloroplasts built with enzymes from *Escherichia coli*, *Chlamydomonas* mitochondria, and pumpkin peroxisomes [10]; folate biofortification of tomato fruit using mouse and Arabidopsis enzymes [11]; the CETCH CO_2_ fixation cycle comprising 17 enzymes from six bacteria, an archaeon, humans, and Arabidopsis [12]; engineering omega-3 long-chain polyunsaturated fatty acid production in Camelina seeds using seven enzymes from chlorophyte and haptophyte algae, two oomycetes, a thraustochytrid, and a moss [13]; and the prospective replacement of plant thiazole synthases with more-efficient ones from near-anaerobic bacteria [14]. Such ingrafted enzymes are a priori unlikely to always be fully active in an alien chassis and some are clearly not fully active [15,16]. Moreover, since pathway engineering failures are far less publishable than successes, mismatches between enzymes and plant chassis likely outnumber the nonsuccesses reported in papers.

## How enzymes can be improved to operate in plants

Enzymes can in principle be improved by rational design, by directed evolution, or by a combination of both (semi-rational design) [17,18]. Rational design is well-suited to improve core enzyme properties, but as it depends on structural and mechanistic data it is not applicable to all enzymes and needs specialist knowledge [17,19]. It is generally not yet suited to the broader task of adapting enzymes to work well in plant platforms (‘plantizing’) beyond mitigating some types of inactivation [20]. In contrast, directed evolution — specifically, *continuous* directed evolution [21] — can improve both core enzyme properties and adaptation to a plant platform, and needs no structural or mechanistic understanding to do so [9,17]. Methods for continuous evolution of metabolic enzymes have recently advanced rapidly [22]. This review outlines these capabilities and advances, with emphasis on the yeast (*Saccharomyces cerevisiae*) OrthoRep system due to its special utility in adapting enzymes for plants.

### Continuous *vs*. classical directed evolution

**Figure 1.**
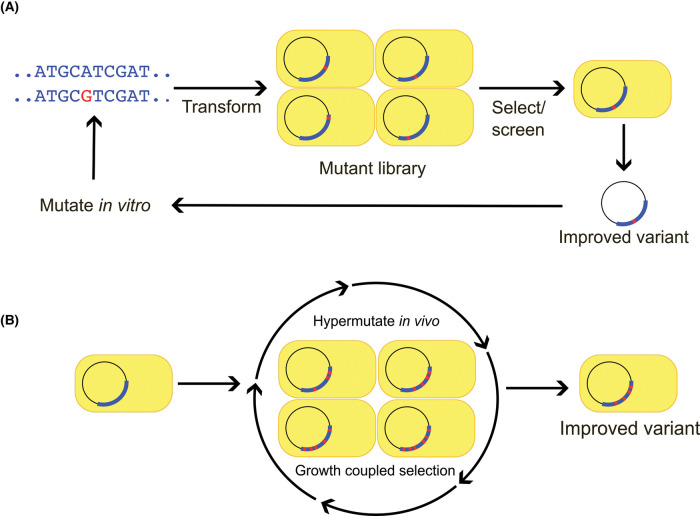
Comparison of classical and continuous directed evolution. (**A**) Classical directed evolution makes mutations (red lines) to the target gene (blue arc) *in vitro*, screens or selects *in vivo*, then re-enters improved variants into the cycle. Each step requires manual intervention. (**B**) Continuous directed evolution subjects the target gene — and only this gene — to a high mutation rate *in vivo*, then selects for improved function *in vivo* via coupling of the target gene function to growth. Once an evolution campaign is started, manual intervention is needed mostly for serial passaging of evolving populations.

Classical directed evolution generates sequence diversity in a target gene *in vitro*, e.g. by error-prone PCR, introduces the resulting library into the host (platform) microbe, screens or selects the clones for improved variants, then repeats this cycle until the desired performance is reached or progress ceases (Figure 1A). Classical systems are bottlenecked by relatively small library size and large demand for manual input, which limit the length and number of the evolutionary paths that can be explored [9,21–23]. Continuous directed evolution systems speed up the workflow by continuously hypermutating the target gene — and only this gene — *in vivo*, and coupling the function of the gene to the growth of the platform cells (Figure 1B); hypermutation can be mediated via an error-prone DNA polymerase (as in OrthoRep) or a nucleobase deaminase fused to T7 RNA polymerase (as in the *E. coli*-based MutaT7 systems) [9,21–23]. Improved variants are obtained simply by passaging cells under selective conditions, with improved target performance manifested as faster growth. The target gene's mutation rate can be 10^5^-fold higher than the natural rate. The large effective library size and small labor demand enable long evolutionary walks to explore multi-peaked fitness landscapes by simultaneously evolving multiple independent populations [21,22]. By using an appropriate multi-well plate format, a campaign can strike a good balance between culture volume and the number of independent populations in high-throughput campaigns. Coupling the function of the target enzyme gene to cell growth can be achieved for many metabolic enzymes by using a platform strain whose corresponding gene has been knocked out, or a strain that has been otherwise manipulated to rely on the target enzyme [6,14,24,25]. Other coupling strategies can also be used, e.g. linking target gene function to an optical output signal for screening via fluorescence-activated cell sorting (FACS) [22]. MutaT7 systems make only transition mutations [26]; the original OrthoRep DNA polymerases make mainly transitions [22] but next-gen OrthoRep polymerases with expanded mutational spectra are expected to become available soon [27].

### Yeast OrthoRep

OrthoRep can be run indefinitely and makes no off-target mutations [24], which is less the case for *E. coli* MutaT7 systems [9]. As a platform to evolve enzymes for use in plants [9], yeast offers eukaryotic redox, protein-folding, proteolysis, and endomembrane systems along with glycosylation and other post-translational mechanisms that most bacteria lack. The absence of plant regulatory mechanisms such as specific residue phosphorylation may be another plus. Furthermore, like plants, yeast is mesophilic, oxygen-tolerant, has mitochondria, and can switch from respiration to fermentation. These general and plant-specific advantages of yeast OrthoRep make it a platform of choice to improve core enzyme characteristics or to plantize enzymes. Evolving an enzyme in a plant platform, while feasible [7], has a far lower throughput than evolution in yeast because of differences in generation times (months *vs*. hours) and population sizes (thousands *vs*. billions). Plantizing in yeast is therefore a far more realistic option, even if conditions in yeast only approximate those in plant cells. For core characteristics such as specificity and kinetics, the approximation should be quite close enough to allow selection for improvements of the desired type and scale that will be retained when the enzyme is transferred to plants.

**Figure 2.**
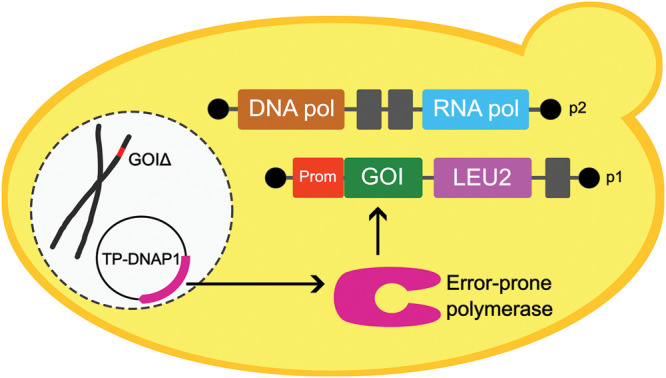
The yeast OrthoRep continuous directed evolution system. In OrthoRep, a nuclear plasmid-borne DNA polymerase engineered to have a high error rate specifically hypermutates a target gene of interest (GOI), inserted behind a suitable promoter (Prom) on the p1 linear cytoplasmic plasmid. This plasmid also carries a *LEU2* selection marker. Another cyto­plasmic plasmid, p2, carries functions needed to express the genes on p1. The platform strain is deleted for the endogenous yeast copy of the gene of interest, which couples growth to the activity of the target gene on p1. There are ∼10 copies of the p1 plasmid per yeast cell.

In OrthoRep (Figure 2), the target gene is expressed from p1, a linear cytoplasmic plasmid that is replicated by an error-prone, p1-specific DNA polymerase borne on a nuclear plasmid. The polymerase can make up to ∼10^−5^ substitutions per base (s.p.b.) in the target gene. A second cytoplasmic plasmid, p2, encodes the RNA polymerase that transcribes genes on p1 and p2. Expression of the target gene can be tuned via the strength of its promoter or via the length of its p1-encoded poly(A) tail, enabling adjustment of the selection window [28]. OrthoRep's power is such that, in *one passage* of a 3 ml culture, *each base* in the target is mutated 3.8 × 10^4^ times [9]. Various selection schemes can be deployed in OrthoRep campaigns, including in a mix-and-match way [9,22]. A simple scheme for an enzyme producing an essential metabolite is to omit the metabolite from the medium; alternatively, its concentration in the medium can be progressively tapered to zero as growth improves. Similarly, the concentration of an inhibitor can be steadily raised. In general, evolution campaigns work best when the selection pressure for the desired enzyme activity can be increased over time. Another generally effective strategy is to start the evolution campaign by growth for ∼100 generations under non-selective conditions so as to build up a large variant library on which selection can then act.

Two potential pitfalls for any continuous directed evolution system are especially important during the long campaigns that OrthoRep makes possible. One pitfall is subclinical contamination; a yeast population can be infected with bacteria yet look and smell normal and show improved growth. Counter­measures include regular checks by plating evolving populations on rich bacterial media and PCR-screening for 16S ribosomal RNA using generic primers. The other pitfall is cheater mutations, which improve growth but occur outside the targeted sequence. Such mutations can be in the nuclear genome or in the target gene promoter on the p1 plasmid (Figure 2). Promoter mutations are easily detected by Sanger sequencing of the p1 plasmid. Wherever a cheater mutation is — promoter or nuclear genome — it can be eliminated from the campaign by recloning the target gene from the evolved population into fresh p1 plasmid in fresh platform cells and re-testing growth under selective conditions.

### Getting improved enzymes into crops

To benefit agriculture, directed evolution must be interfaced with plant breeding programs. Three ways to do this are as follows, ordered from the least to the most desirable. First and worst comes transgenesis. Although long-established technology, inserting transgenes and regenerating plants is still not routine, or even possible, for many crops [29] including important horticultural ones (e.g. [30,31]). Furthermore, transgenic technology can require screening a large number of transgenic events to obtain phenotypically normal lines with stable transgene expression, making it costly. Transgenics also remain highly problematic for regulators and consumers in many major markets, particularly for horticultural crops [32]. A second way to turn the output from a directed evolution campaign into the input for a breeding program is to combine directed evolution with genome editing (DE-GE) [33]. Directed evolution identifies mutations that improve the function of a target plant enzyme; the base changes needed to alter the amino acid sequence of the endogenous target enzyme are then introduced via CRISPR gene-editing technology. Although CRISPR gene-editing is still largely reliant on, and bottlenecked by, transformation and plant regeneration [29], DNA-free and transformation-free editing methods now exist [31,34] and the final line can in any case be transgene-free and hence more favorable for public acceptance if the CRISPR machinery is bred out using conventional breeding [34]. A third way is again to use directed evolution to identify beneficial mutations in a plant enzyme, to scan crop and crop-relative genomes for precisely those mutations, and then to introduce the mutations into the crop by molecular breeding methods. The new FIND-IT (Fast Identification of Nucleotide variants by droplet DigITal PCR) approach [35] could speed up the scanning step for targeted enzyme variants. FIND-IT is species-independent and applicable to low-mutation density variant populations from natural germplasm as well as from induced mutagenesis [35]. FIND-IT should therefore be suitable for blueberries [36], strawberries [37], and other horticultural crops with massive allelic diversity, or alternatively for large variant libraries generated by chemical mutagens if there is too little natural variation. Note, however, that high allelic diversity within target regions may complicate the detection of desired mutations, especially for polyploid crops. Note also that searching for desired variants in existing germplasm is favored by the fact that single-base-pair editing remains inefficient and is not yet available for most crops [31].

## What directed evolution can do in horticultural crop breeding

Numerous recent reviews and perspectives have covered how synthetic biology in general and directed evolution in particular could help agriculture, e.g. [33,38–48]. With a few exceptions such as Rubisco [47] and certain short-lived enzymes [48], these articles have not tried to look forwards by pinpointing specific traits of particular enzymes that are technically feasible to improve and likely to be agriculturally relevant. They have instead looked backwards to a small set of iconic examples and taken a broad-brush approach to the future that is envisioned in bold but vague terms like ‘optimizing’, ‘redesigning’, ‘tailoring’, ‘efficiency’, ‘targeted elimination of undesired traits’, ‘high-value compounds’ and ‘collaboration with Nature’. An emerging consensus holds that it is time to move past generalities and to engage with specific enzyme targets for crop improvement that have a reasonable change of working at scale and doing so in years rather than decades or centuries [49]. The section below sets out to do this in the context of horticulture. We first delimit what is within directed evolution's reach and what types of trait breeders need, then look at what falls in the overlap between these two spaces.

### What directed evolution can do

A directed evolution campaign can basically improve one enzyme-encoding gene at a time, or a small number of genes such as those for a multi-subunit enzyme [50]. Campaigns usually culminate after introducing a fairly small number of mutations because just one or a few amino acid changes often trigger new properties and thereafter diminishing returns set in [51,52]; nearly all campaigns stop before 20 synonymous mutations and most stop before 10. As said above, the target enzyme must have a selectable or screenable activity, and in the case of OrthoRep, only enzymes whose activity can be coupled to growth are convenient targets.

**Figure 3.**
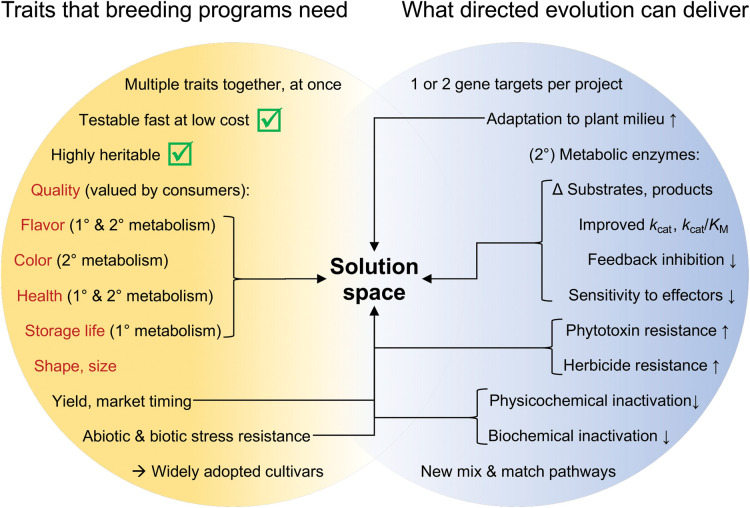
Mapping what directed evolution can do onto what breeding programs need. The left-hand circle shows how fruit and vegetable breeding programs work to balance multiple traits simultaneously, that they prefer highly heritable traits that can be screened quickly and cheaply, and that the most important traits are typically aspects of quality (in red font, valued by consumers) as well as production traits such as yield, stress resilience, and herbicide resistance. The end goal is to release successful cultivars. The right-hand circle shows the capabilities of directed enzyme evolution, particularly in the OrthoRep system. Where the two circles blend is the Solution Space in which breeding problems that really need solving meet solutions that directed evolution can really provide. The green check marks in the left-hand circle emphasize that the enzyme traits improved by directed evolution are intrinsically highly heritable and readily tracked by molecular breeding methods since they are specified by one or a very few defined genes. 1°, primary metabolism; 2°, secondary metabolism.

Thus directed evolution's scope is limited in practice to enzymes encoded by just one or two genes, to improvements that lie within a few mutations of the starting point, and — for OrthoRep — to activities upon which yeast cells can be forced to depend. Although seemingly small, this scope in fact encompasses a large range of metabolically and agriculturally important characteristics, as we sketched in the opening section and cover here and in Figure 3 in more detail. In rough order of research and development effort so far, first are substrate and product specificity, i.e. the input molecules, natural or synthetic, that an enzyme can convert into desired output molecules [2] and how many mistakes it makes in the process [53]. Next are kinetic features — *k*_cat_ and *K*_M_ values — which dictate how much enzyme is needed to convert substrate to product at the desired rate [54,55]. Then come feedback inhibition by pathway end-products [6], and sensitivity to other endogenous effectors [56] and to exogenous chemicals such as natural small-molecule phytotoxins and herbicides [57–59]. Last come resistance to inactivation by temperature [60], oxygen [14], or the catalytic reaction itself [20], and overall adaptation to a plant milieu [9].

A higher-order limitation on the scope of directed enzyme evolution is the target enzyme's natural or designed position in the metabolic network. Primary metabolic enzymes have been subjected to longer and stronger selection for specificity and high flux capacity than secondary metabolic enzymes, making primary metabolic enzymes harder to improve, especially with respect to action on their physiological substrates [5,48]. Furthermore, primary metabolism has multiple layers of enzyme regulation and enzyme–metabolite interactions that, in whole plants — especially under field conditions — can subvert or nullify an engineered improvement [61]. Enzymes of secondary metabolism are overall more tractable targets because they evolved relatively recently, and are broad-specificity, slow, low-flux catalysts [62].

### What breeding programs need

Breeding fruit and vegetable crops nearly always requires selecting simultaneously for multiple traits [63]. The traits can be roughly split into grower-oriented production traits such as yield and resilience, and consumer-oriented quality traits like flavor, color, size and shape, retention of freshness, and value to health, of which flavor and health benefits are now particularly important [63–67] (Figure 3). The need to keep track of multiple traits puts a premium on those that are highly heritable and can be screened reliably, quickly, and cheaply [63]. Genes improved by directed evolution meet both criteria because (i) as single loci they are highly heritable, and (ii) nucleotide polymorphisms in plant genomes can be readily sequenced. Once an improved target gene is obtained from a directed evolution campaign and introduced into a breeding population, it is thus in principle straightforward to track by molecular breeding methods [68]. The challenge is to choose a useful and feasible target enzyme gene in the first place, i.e. one that falls in the Solution Space of Figure 3, as we explain next.

### The Solution Space: accessible enzyme traits that are worth breeding for

It would be poor intellectual property management to identify specific target enzyme traits at this point, but Figure 3 plus one overarching principle are enough to define general areas with good targets. The overarching principle [69] is that the enzyme trait must have a large, dominant effect that scales readily from the molecular to the whole-plant and crop levels. The classic single-transgene insect resistance and herbicide tolerance traits are cases where phenotype scales directly from molecular-level action [70]; modifications to photosynthesis (e.g. [10]) are cases where it may well not [69,71].

Horticultural breeding priorities and directed evolution capabilities overlap strongly in quality traits. As the arrows in Figure 3 show, flavor, color, health benefits, and storage life/spoilage are heavily influenced or even fully determined by the activities of primary and secondary metabolic enzymes. Examples of fruit cultivars with enzyme-governed traits include: The long shelf-life Flavr Savr^TM^ tomato (enzyme: polygalacturonase), available 1994–1997; the nonbrowning Arctic® apple (enzyme: polyphenol oxidase), which debuted in 2017; and the lycopene-enriched Pinkglow^TM^ pineapple (enzymes: phytoene synthase, lycopene β-cyclase, and lycopene ε-cyclase), released in 2020 [72]. These prior examples all used transgenesis and RNAi technology, but the same phenotypes could now in principle be obtained by directed evolution and genome editing more precisely, and perhaps less contentiously. The fast-growing knowledge of phytochemical diversity, of the determinants of organoleptic quality and health benefits, and of horticultural crop genomes [64–68,72–74] is opening up many enzyme-mediated quality traits for improvement, using native or non-native enzymes. The latter can if necessary be plantized using OrthoRep [9] (Figure 3). Active health-centric areas include modifying starch synthesis and deposition to lower the glycemic index [75], natural non-sugar sweeteners [76], and prebiotics [77].

Production traits that hinge on resistance to an exogenous compound are another area where horticultural crop breeding and directed evolution overlap (Figure 3). When the exogenous compound is a herbicide that inhibits a particular enzyme, the enzyme can often be evolved to reduce inhibition, conferring herbicide-resistance at the crop level. The DE-GE approach, i.e. evolving a crop's own enzyme gene and then replacing the native gene with the evolved one, is well-suited to this [6,33]. Such an approach could be particularly useful in horticultural crops, for which weed-control options are generally limited [78]. A similar strategy could be deployed to increase resistance to a phytotoxin made by a pathogen when the phytotoxin inhibits a specific enzyme [79].

Cell wall-related traits cross over between production and quality; these, too, are amenable to directed enzyme evolution. Provided that they can be coupled to growth or probes for fluorescence-activated cell sorting, carbohydrate-active enzymes could be improved in yeast for subsequent use in plants [80]. For example, enzymes that produce more viscous polysaccharides are desired to increase the satiety of plant-based foods and reduce blood glucose spikes. Directed evolution campaigns could thus bypass the decades-long challenge of predicting substrate specificity from the structure of enzymes that transfer or hydrolyze glycans. Cell wall metabolism enzymes influence fruit shape, size, and suitability for mechanical harvesting, post-harvest shelf life, and soluble/insoluble fiber content. A clear precedent comes from the Flavr Savr^TM^ tomato mentioned above, where the cell wall enzyme polygalacturonase was the target. A recent success was the use of FIND-IT to identify single nucleotide polymorphisms that reduce mixed-linkage glucan in elite barley lines (desired for malting quality; enzyme: Cellulose Synthase-Like F6, CSLF6) [35]. While most CSLF6 mutations reduced yield or compromised grain integrity during threshing, a CSLF6 variant producing intermediate mixed-linkage glucan levels maintained total grain yields and has been commercialized [35].

### Actionable conversations

Directed enzyme evolution is the domain of biochemists. Crop improvement is the domain of breeders and agronomists. While biochemists, breeders, and agronomists talk the common language of genetics, beyond that they differ in many ways ranging from worldview (reductionism *vs*. holism) and biological interactions studied (molecular *vs.* genotype × management) through project length (days to months *vs*. years to decades) to performance criteria (publications *vs*. cultivars *and* publications). Moreover, breeding programs are essentially a set of interconnected hub-and-spoke operations in which the breeder is the hub, supported by a set of spokes among which the biochemist is just one. Other spokes include pathologists, food scientists, soil scientists, industry stakeholders, and consumers. Deploying directed evolution effectively in a breeding program therefore demands mutual acculturation. This takes time but it eventually leads to actionable conversations in which aims are defined so that experiments start, grant proposals get written, and products are delivered to consumers. Horticulture has a long, productive tradition of such intradisciplinary dialog, as does agricultural biotechnology [81]; for horticultural crops, the ongoing two-way exchanges between post-harvest science and the biochemistry and physiology of ethylene [82] and gibberellins [83] are excellent examples.

## Perspectives

- Continuous directed evolution in the yeast OrthoRep system can be used both to improve various aspects of plant enzyme function and to adapt nonplant enzymes to work well in plants.
- Many recent articles express justified optimism about prospects for deploying directed evolution in crop improvement, but only identify target traits in vague terms such as ‘yield’ or ‘resilience’.
- It is now urgently necessary for biochemists and plant breeders to jointly identify target enzyme traits that consumers or producers want and that directed evolution could substantially improve, and to work together to start integrating these traits into breeding programs.

## Open Access

Open access for this article was enabled by the participation of University of Florida in an all-inclusive *Read & Publish* agreement with Portland Press and the Biochemical Society under a transformative agreement with Individual.

## Abbreviations

DE-GE: directed evolution with genome editing
FIND-IT: Fast Identification of Nucleotide variants by droplet DigITal PCR
